# The Development and Evaluation of a New Inactivated Vaccine against *Mycoplasma capricolum* subsp. *capricolum*

**DOI:** 10.3390/microorganisms12061118

**Published:** 2024-05-31

**Authors:** Noha Semmate, Zahra Bamouh, Zouhair Elkarhat, Soufiane Elmejdoub, Mazen Saleh, Ouafaa Fassi Fihri, Mehdi Elharrak

**Affiliations:** 1 Department of Pathology and Veterinary Public Health, Agronomic and Veterinary Institute Hassan II, BP 6202, Rabat-Instituts, Rabat 10101, Morocco; z.bamouh@mci-santeanimale.com (Z.B.); o.fassifihri@iav.ac.ma (O.F.F.); 2 Department of Research and Development, Multi-Chemical Industry Santé Animale, P.O. Box 278, Mohammedia 28810, Morocco; z.elkarhat@mci-santeanimale.com (Z.E.); s.elmejdoub@mci-santeanimale.com (S.E.); m.elharrak@mci-santeanimale.com (M.E.); 3 Institute of Thematic Research Specialized in Medical, Food and Environmental Biotechnology of Benslimane, Hassan II University of Casablanca (UH2C), Casablanca 20670, Morocco; 4 School of Natural Sciences, Laurentian University, Sudbury, ON P3E 2C6, Canada; and the Northern Ontario School of Medicine University, Sudbury, ON P3E 5Z6, Canada; msaleh@laurentian.ca

**Keywords:** *Mycoplasma capricolum* subsp. *capricolum*, *Mycoplasma capricolum* subsp. *capripneumoniae*, vaccine, contagious agalactia, contagious caprine pleuropneumonia

## Abstract

*Mycoplasma capricolum* subsp. *capricolum* (Mcc) and *Mycoplasma capricolum* subsp. *capripneumoniae* (Mccp) are pathogens that affect large and small ruminants. Indeed, Mcc affects both sheep and goats, causing contagious agalactia (CA). Mccp affects only goats, causing contagious caprine pleuropneumonia (CCPP). CA and CCPP are mainly controlled using inactivated Mcc and Mccp vaccines. However, producing the vaccine with the Mccp strain is complex, fastidious, and costly due to the slow growth of the bacterium. In this study, we present new oil-adjuvanted and inactivated Mcc and Mccp vaccines for sheep and goats against CA and CCPP. The vaccines were evaluated for safety and efficacy using experimental infection. A serological response was observed one week after of the first vaccination of sheep and goats with Mcc and goats with Mccp. The vaccinated animals were subsequently challenged with the virulent Mcc MOR20 strain. The Mcc vaccine was demonstrated to provide robust protection when the animals were challenged with Mcc MOR20. Cross-protection against the Mcc MOR20 challenge was also obtained with the Mccp vaccine. This finding revealed, for the first time, the safety and efficacy of an inactivated Mcc vaccine against contagious agalactia and cross-protection between Mcc and Mccp strains.

## 1. Introduction

*Mycoplasma capricolum* species includes two major subspecies, *Mycoplasma capricolum* subsp. *capricolum* (Mcc) and *Mycoplasma capricolum* subsp. *capripneumoniae* (Mccp). Both pathogens have a significant negative impact on the small ruminant production chain and result in substantial economic losses [1]. Mcc and Mccp share a high degree of genetic similarity [2] and can produce respiratory symptoms in goats which can generate confusion in the diagnosis and control of infectious diseases [3]. In addition to a respiratory syndrome, Mcc is one of the etiological agents of contagious agalactia (CA), a disease that causes arthritis, keratoconjunctivitis, pneumonia, and mastitis in lactating animals and septicemia and abortions in pregnant females [4]. 

CA imposes considerable economic losses due to reduced milk production, mortality in young animals, abortion, and expenses incurred in treating affected animals [5]. In addition to Mcc, other etiological agents of CA could be *Mycoplasma agalactiae*, *Mycoplasma mycoides* subsp. *capri*, and *Mycoplasma putrefaciens* [6]. The management of CA is particularly difficult due to the limited efficacy of antibiotics and because the use of antimicrobial therapy during the weaning period results in a decrease in milk production. Moreover, residues of antimicrobials in milk present potential health risks for consumers. Thus, vaccination remains the most effective tool for controlling CA in small ruminants [7]. 

Mcc is the main etiological agent responsible for CA in Morocco, according to several studies [8,9,10]. Despite the high prevalence of the disease and its high pathogenicity, no vaccination program has been carried out in North Africa, and no publications can be found that report on the use of such vaccines in small ruminants. In this study, we present, for the first time, data on protection obtained through the use of an inactivated vaccine against Mcc in sheep and goats. The safety and efficacy of the vaccine were assessed by monitoring serological responses and experimental infection. Indirectly, the performed experiments assessed cross-protection between Mcc and Mccp.

## 2. Materials and Methods

### 2.1. Bacteria

Two strains of *Mycoplasma capricolum* were used for vaccine production. The strain *Mycoplasma capricolum* subsp. *capricolum* (Mcc) MOR20, isolated in Morocco in 2019 [8], was used for vaccination and challenge. *Mycoplasma capricolum* subsp. *capripneumoniae* (Mccp) strain F38, originally isolated in Kenya in 1970 [11], was used to prepare the Mccp vaccine.

### 2.2. Vaccine Preparation

The two strains were cultured in a PPLO broth (BD, Franklin Lakes, NJ, USA) medium supplemented with 20% equine serum (Serox, Mannheim, Germany), 10% fresh yeast extract (Solabia, Saint-Beauzire, France), and 1% glucose and incubated under the same conditions, 37 °C with 5% CO_2_. Mycoplasma titration was carried out using the standard method of microtitration and color change, and the titer was calculated using the Spearman–Karber formula and expressed as CCU5, color-changing units [12]. Inactivation was performed using binary ethylenimine (BEI) (Sigma-Aldrich, St. Louis, MO, USA) at a concentration of 0.01 M. Antigens were then concentrated by ultrafiltration (500 Kda). Protein quantification was performed using bovine serum albumin as a reference standard (WOAH, 2000) [13]. 

### 2.3. Vaccine Formulation

Four inactivated Mcc vaccines were formulated using three different adjuvants mixed with an antigen of Mcc at a dose of 0.3 mg per animal. Vaccine 1 contained the inactivated suspension of Mycoplasma with a Montanide adjuvant (Seppic, Paris, France) at a ratio of 4:6 in an emulsion of water in oil (W/O). Vaccine 2 contained the inactivated suspension of Mycoplasma with a purified saponin at a final concentration of 3 mg/mL, as recommended in the WOAH Manual (CHAPTER 3.8.4) [13]. Vaccine 3 contained the inactivated suspension of Mycoplasma with a Montanide adjuvant (Seppic, France) at a ratio of 85:15 in an emulsion of oil in water (O/W). Vaccine 4 contained an inactivated suspension of Mccp at a dose of 0.15 mg per animal with a Montanide adjuvant (Seppic, France) at a ratio of 4:6 in an emulsion of water in oil (W/O) (Table 1).

### 2.4. Vaccination Protocol

Sixteen sheep and thirty-two goats, 4–6 months old, which tested negative for Mcc and Mccp antibodies via an ELISA were housed in an ABSL3 facility. Animal experiments were carried out in accordance with international guidelines for the care and handling of experimental animals, as described in a protocol approved by “The MCI Santé Animale Ethic Committee for Animal Experiment”. The first experiment involved 16 sheep divided into four groups (S) of 4 animals each (Table 2). S1 was vaccinated with an Mcc W/O-based formulation, S2 was vaccinated with Mcc formulated with a saponin, and S3 was vaccinated with an Mcc O/W-based formulation. Group S4 comprised unvaccinated control sheep. The second experiment included 12 goats divided into three groups of 4 goats (Table 3). G1 was vaccinated with the Mcc, and G2 was vaccinated with the Mccp; both vaccines were W/O-based vaccines. G3 was the unvaccinated control group. Vaccination was performed by the administration of 1.0 mL of the corresponding vaccine at day 0 and a secondary vaccination at day 28 pv via the subcutaneous (SC) route for the saponin and O/W vaccines (S2 and S3) and via the intra-muscular (IM) route for the W/O vaccines (S1, G1, and G2), as recommended by the adjuvant supplier.

### 2.5. Evaluation of Antibody Response

Blood samples were collected weekly until D42 pv from all animals (goats and sheep). Sera were extracted and submitted for a serology analysis by an enzyme-linked immunoassay as described by Semmate et al. [8]. Briefly, this experiment involved the use of microplates coated with an Mcc lysate. The Mcc antigen was immobilized in a bicarbonate buffer within each well of a Maxisorp plate and allowed to incubate overnight at 4 °C. Following this, the plate underwent washing with phosphate-buffered saline (PBS). Subsequently, sera collected from the goats and sheep, suitably diluted in blocking buffer, were added to the plates. After a 1 h incubation at 37 °C, the plate was again washed with PBS containing Tween 20 (Sigma-Aldrich, St. Louis, MO, USA). Next, it was incubated with horseradish peroxidase-conjugated anti-immunoglobulin G (obtained from Bethyl Laboratories, Montgomery, MD, USA) for an additional hour at 37 °C. The plate underwent another washing step with PBS containing Tween, and the substrate 3,3′,5,5′-tetramethylbenzidine dihydrochloride (Medicago, Uppsala, Sweden) was added, reacting for 10–15 min at room temperature. The enzymatic reaction was terminated by the addition of 50–100 μL of sulfuric acid (Sigma, St. Louis, MO, USA) per well, and the optical density was measured at 450 nm. The assessment of specific Mcc or Mccp antibodies was based on optical density as an indicator of antibody presence. For the detection of Mccp antibodies, a commercial kit (IDEXX, Paris, France) was first used, and it was then replaced by the Mcc ELISA described above after validation.

### 2.6. Experimental Mcc Infection of Sheep

Two weeks after the second vaccination (D42), the 4 sheep groups of the first experiment, S1, S2, and S3, were challenged by administering 10 mL per animal of the virulent Mcc MOR20 strain at a dose of 10^8^ ccu/mL via the intratracheal route; inoculations were performed daily on D1, D2, D3, and D4 [14]. The sheep were monitored daily for rectal temperature and clinical symptoms for a one-month period post infection (pi). Clinical scoring was conducted according Semmate et al. [8] (Table 4). Blood samples were taken in dry tubes from all animals weekly during the one-month pi period for an ELISA. Autopsies were performed on euthanized animals that displayed signs of distress and surviving goats at the end of the study. The animals were anesthetized by an intravenous administration of xylazine (Rompun 2% Bayer, Leverkusen, Germany) and an intramuscular administration of ketamine (Imalgene 1000, Boehringer Ingelheim, Ingelheim am Rhein, Germany).

### 2.7. Experimental Mcc Infection of Goats

Two weeks after the second vaccination (D42), four goats from each group of the second experiment were challenged by administering 1.0 mL of the virulent Mcc MOR20 strain at a dose of 10^8^ ccu/mL per animal via the subcutaneous route [15]. The goats were monitored daily as described above for sheep.

## 3. Results

### 3.1. Mcc/Mccp Vaccine Production and Inactivation

**Figure 1:**
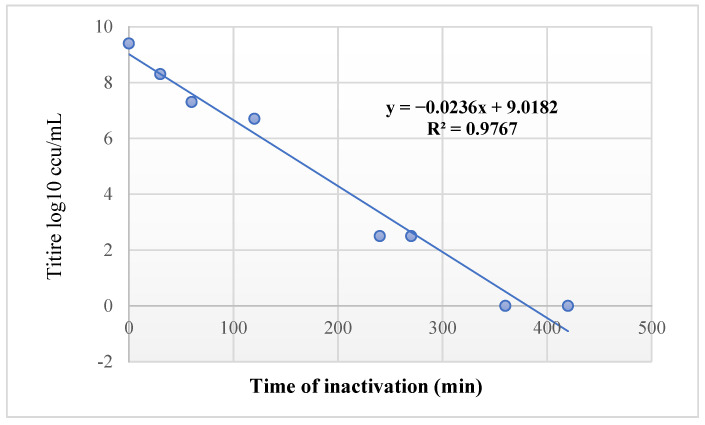
Inactivation kinetic of Mcc MOR20 strain using 0.01 M BEI.

For the antigen preparation, the optimal titer for Mcc was 10^9.9^ ccu/mL, and it was obtained after 24 h of culture, whereas the titer of Mccp was 10^9^ ccu/mL after 7 days of culture. Harvested suspensions of the two strains were inactivated using BEI after 7 h (Figure 1). The oily vaccine formulation resulted in a white-colored emulsion, with one phase of a water-in-oil type or an oil-in-water type. The vaccine containing the saponin resulted in an aqueous grey transparent phase.

### 3.2. Safety of the Mcc Vaccines in Sheep

Following the first injection, 2/4 sheep in the S1 group (W/O) and S3 group (O/W) showed 3-day moderate hyperthermia (from D1 to D3 pv) and, after the second injection, the same two animals showed one day of hyperthermia (40 °C). Sheep vaccinated with saponin-adjuvanted vaccine S2 all reacted with a fever reaching 40.7 °C from D1 to D4. Moderate hyperthermia was observed in three sheep after the booster in this saponin group. No local reaction was noted after the first vaccination of sheep group S1, while sheep vaccinated in the S2 and S3 groups presented local inflammation at the injection site (4 and 2 cm in diameter, respectively) for two weeks starting at D4 pv. At the second injection, no local reaction was detected in either group.

### 3.3. Safety of Mcc and Mccp Vaccines in Goats

All goats remained healthy without any adverse reaction after vaccination. Moderate hyperthermia was noted in the G1 vaccinated group after the first Mcc injection, lasting 2 days at 40 to 40.3 °C. No fever observed after the booster. In group G2 vaccinated with Mccp, 3-to-5-day hyperthermia (40.1 °C) was noted in three out of four goats. Following the booster injection, hyperthermia was noted in two of the four goats. No local reactions were recorded after the first and second vaccinations in either group.

### 3.4. Evaluation of Antibody Response to Vaccination

**Figure 2:**
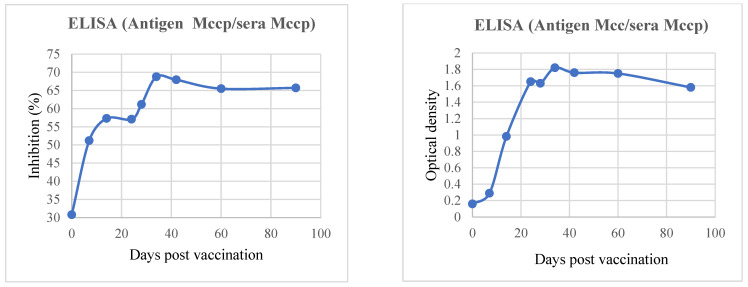
Antibody response in goats vaccinated with Mccp and Mcc.

**Figure 3:**
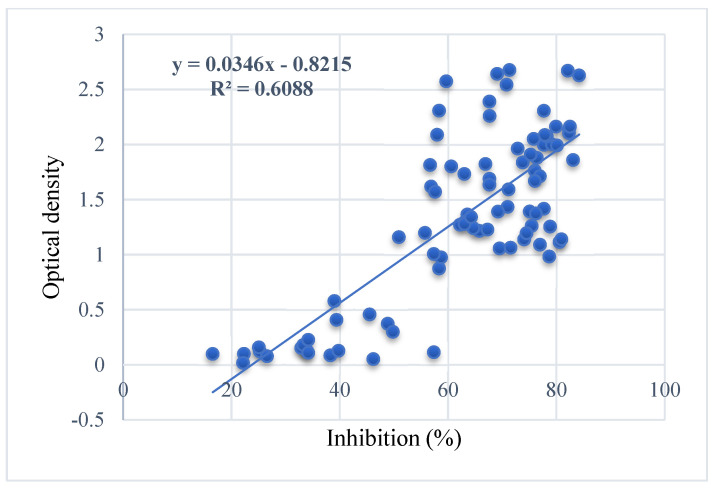
Correlation between Mcc (homemade) and Mccp (IDEXX) ELISAs.

The homemade Mcc ELISA antibody results were compared to those obtained using the commercial Mccp competitive ELISA to validate the immunological response to vaccination. As reported in Figure 2, the Mcc indirect ELISA can indiscriminately detect Mcc and Mccp and vice versa. There is a close positive correlation between the results of the two ELISAs (R: 0.78024), as reported in Figure 3.

### 3.5. Antibody Response of Sheep to Vaccination

**Figure 4:**
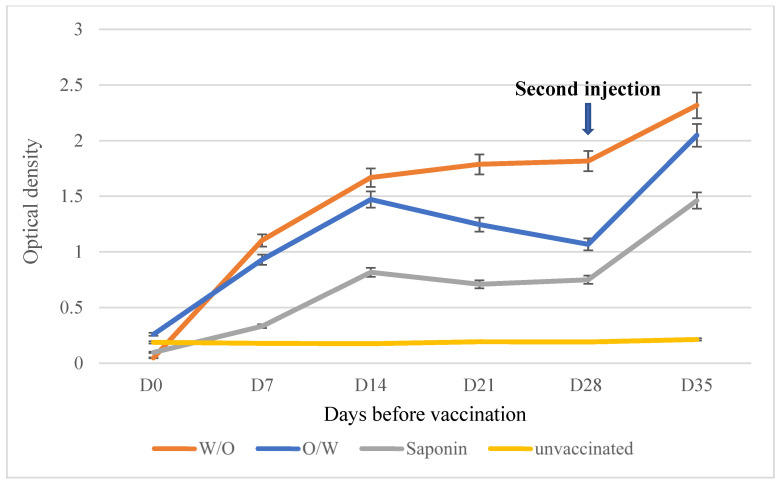
ELISA antibody response in sheep immunized with: Mcc W/O (S1), Mcc saponin (S2) and Mcc O/W (S3) vaccines.

All vaccinated sheep from the S1, S2, and S3 groups had positive ELISA antibody test results at D7 pv, and titers peaked on D14 pv after the first injection, with a clear difference between the oil-adjuvanted vaccines and the saponin aqueous vaccine. After the second vaccination, a significant increase in antibody titers was observed in the three groups. Sheep in the S1 group vaccinated with the W/O vaccine showed significantly higher antibody responses than the sheep vaccinated with the aqueous vaccine or the O/W vaccine (Figure 4). 

### 3.6. Serological Response of Goat to Vaccination

**Figure 5:**
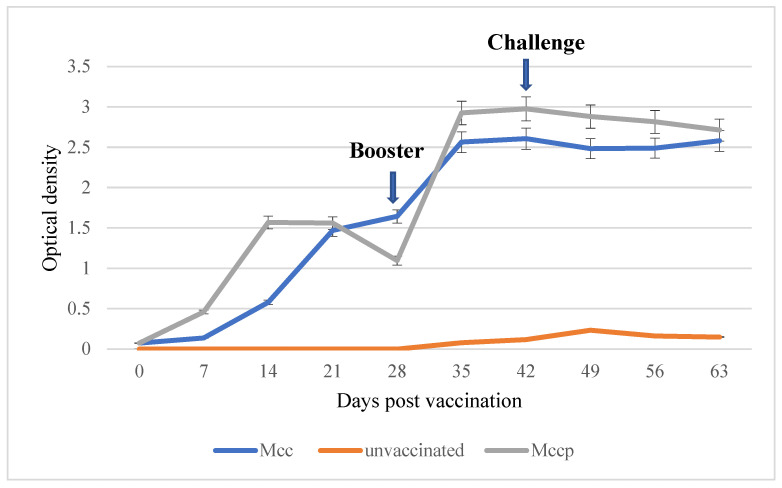
Average Mcc and Mccp antibody responses after vaccination and challenge.

All goats from G1 (vaccinated Mcc) seroconverted, with an increase in antibody titers at D35 after the booster. Initially the titer was 0.07 at D0, and it reached 1.6 at D28 and 2.5 after the booster. Goats from G2 also seroconverted, reaching a titer of 2.9 at D35 after the second injection of the Mccp vaccine. No increase in antibody titers was observed after the Mcc challenge in either G1 or G2 (Figure 5).

### 3.7. Clinical Observation and Lesions Post Challenge in Sheep

After the challenge, one of four unvaccinated sheep presented typical symptoms of Mcc infection (715), including lameness, diarrhea, nasal discharge, anorexia, and keratoconjunctivitis, and died on D12 pi. At necropsy, the animal presented hypertrophy of prescapular lymph node, inflammation, and necrotic tissue in its joints (Table 5). Three other unvaccinated animals showed lameness, cough, and nasal discharge, with an absence of macroscopic lesions. 

**Figure 6:**
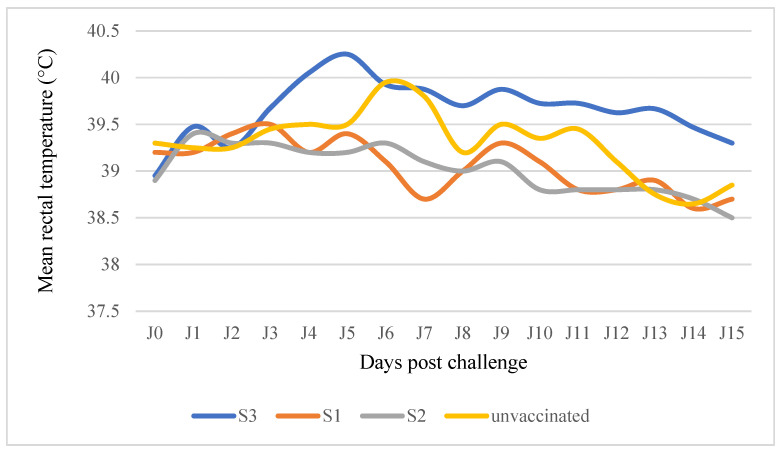
Average body temperature post challenge in sheep vaccinated with Mcc vaccines. S1 Mcc adjuvanted with W/O, S2 adjuvanted with saponin, and S3 adjuvanted with O/W.

Sheep vaccinated with the Mcc W/O vaccine (S1 group) vaccine showed only a slight temperature after the challenge (Figure 6), and in the S2 group vaccinated with the Mcc saponin vaccine, one animal showed lameness and two animals showed nasal discharge and hyperthermia. In the S3 group vaccinated with the Mcc O/W vaccine, two animals presented lameness, fever, and nasal discharge. At necropsy, no characteristic lesions were observed in the vaccinated animals, and only light hypertrophy of the prescapular lymph node was noted in two sheep in group S3.

### 3.8. Clinical Observations and Lesions Post Challenge in Goats

**Figure 7:**
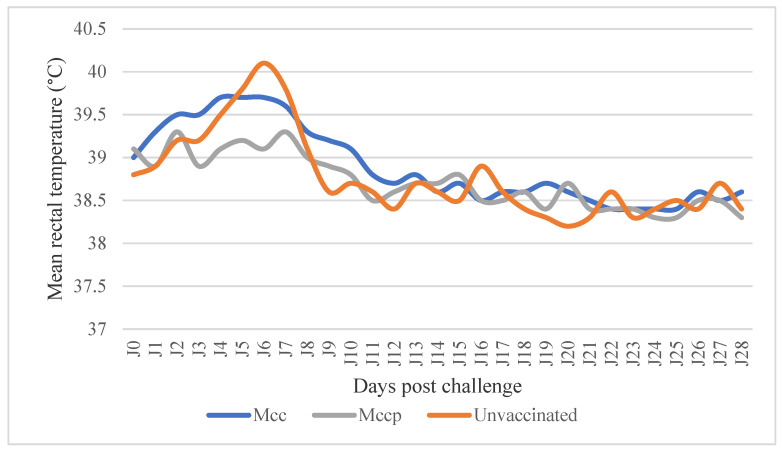
Average body temperature post challenge in goats vaccinated with Mcc and Mccp inactivated vaccines.

After the challenge, three of four unvaccinated goats showed hyperthermia (D3 and D8 pi); 5-day hyperthermia was observed in animal 933 (peak: 41, 1 °C), 4-day hyperthermia was observed in goat 420 (peak: 40, 1 °C), and 3 days of hyperthermia was observed in goat 405 (peak: 40, 5 °C) (Figure 7). Two goats presented typical symptoms of Mcc infection (933and 405), including lameness, diarrhea, nasal discharge, anorexia, and keratoconjunctivitis, and they died on D12 and D13 pi with clinical scores of 23 and 24, respectively (Figure 7). At necropsy, goat 933 presented specific lesions including lung consolidation, intestinal congestion, hypertrophy, and necrosis of the pulmonary lymph node and inflammation and necrotic tissue in joints.

In Mcc-vaccinated goats from G1, moderate hyperthermia was recorded in animal 422 between D3 and D7 pi. Mild clinical symptoms such as nasal discharge and cough were noted in three goats. At necropsy, goat 422 presented hypertrophy of the prescapular lymph node. The mean clinical score of this group was 3 (Table 6). In goats from the G2 group vaccinated with Mccp, no hyperthermia was recorded after the challenge. However, goat 902 presented slight symptoms including nasal discharge and cough, with a mean clinical score of 2.25 for the group (Table 5). At necropsy, no characteristic lesions were observed, only light hypertrophy of the prescapular lymph node in two goats.

## 4. Discussion

As highlighted by the World Organization for Animal Health (WOAH), Mycoplasmas are responsible for economically important diseases of sheep and goats, such as contagious caprine pleuropneumonia (CCPP) caused by *Mycoplasma capricolum subsp. capripneumoniae* (Mccp) and contagious agalactia (CA) caused by *Mycoplasma capricolum subsp. capricolum* (Mcc), among other species [13]. Economic consequences affect various aspects of goats and sheep farming, including livelihood production and the export of animal products worldwide. It is therefore essential to develop effective control strategies to prevent these diseases and reduce financial losses [5]. For CA, vaccination is not commonly practiced despite the significant economic impact of the disease in the production chain of small ruminants. This is further complicated by the lack of published research on vaccine development and evaluation. For CCPP, several types of vaccines were developed, including inactivated, live, sub-unit, and recombinant vaccines [16]. Only inactivated and live vaccines are commercially available [7].

In 2019, a new Moroccan strain of Mcc was isolated and characterized from outbreaks of CA in goats [8]. A genetic analysis of Mcc MOR20 based on LppA gene sequencing showed 97% similarity with the Mcc California strain originating from goats and 71% similarity with the Mccp cluster. Experimental infection induced a severe and generalized disease in both adults and young small ruminants [8]. Because Mcc MOR20 represents a potential vaccine strain against CA, the aim of this study was to evaluate the safety and the potency of a new inactivated vaccine against Mcc in sheep and goats. The vaccine was formulated with three different adjuvants and evaluated by serology response and challenge. In addition, cross-protection between Mccp and Mcc was estimated through a comparative vaccination of goats with Mcc and Mccp vaccines. 

In this study, the growth kinetics of Mcc and Mccp had the same conditions, and the results showed significant advantages of the use of Mcc in production rather than Mccp if we consider the yield obtained; after 24 h of culture, Mcc had already reached the stationary growth phase vs. the 7 days required for Mccp. An inactivation technique based on BEI was recommended by Rodríguez et al. in 2004 [17] and carried out for several mycoplasmas, including Mcc, and it has demonstrated its effectiveness without compromising immune proteins. 

Vaccines containing a saponin as an adjuvant have several limitations, such as the induction of transient antibody responses requiring frequent revaccination at 6- or even 4-month intervals [18,19]. In contrast, oil-based-adjuvant vaccines have been shown to induce a robust, long-lasting antibody response, resulting in enhanced efficacy [20,21]. This study carried out a comparative evaluation between a saponin-based adjuvanted vaccine and a vaccine using Montanide as an adjuvant in two emulsion types, W/O and O/W. 

Regarding safety, the oily vaccine with a W/O adjuvant, injected via the IM route, did not produce any visible reaction in contrast to the saponin-based and oily O/W-emulsion vaccines, which induced local inflammation and fever when injected via the SC route. 

In our study, antibody response was assessed using ELISAs, a CCPP commercial kit and a homemade Mcc test. The results showed that both kits detected the same level of antibodies against Mcc and Mccp, and we cannot differentiate between the two diseases using serology via the local or commercial ELISA kit. This is in accordance with Ruffin et al., 2001 [3]. 

The Mcc vaccine adjuvanted with a W/O emulsion induced a significantly higher antibody response in sheep than the saponin-based vaccine. This observation highlights the superiority of an oil adjuvant in comparison to a saponin adjuvant as the same quantity of antigen was used in both vaccines. This response was significantly reinforced after the second injection of the vaccine, giving evidence that the saponin-adjuvant vaccine requires two injections. However, two sheep vaccinated with the O/W vaccine showed typical CA symptoms, although their antibody response was good, leading us to deduce that the ELISA antibody response does not correlate well with protection. The same results were shown in the CA vaccination trial by Tola et al. in 1999 [22]. We conclude that in this first experiment, the highest level of protection was obtained from the W/O vaccine followed by the saponin-based vaccine, while the O/W vaccine is not protective for sheep against Mcc infection.

In the second experiment, all goats seroconverted after vaccination in contrast with Matios et al. [6], who reported seroconversion in only 61.1% of goats vaccinated with an inactivated Mccp vaccine adjuvanted with saponin. This low seroconversion rate could be explained by the use of an insufficient Mccp antigen or by the adjuvant used in the vaccine [23]. Serology post vaccination and post challenge showed that goats respond earlier to CCPP vaccination and infection compared to their Mcc response, which is probably due to higher goat specificity to CCPP mycoplasma. This hypothesis is supported by the challenge results as unvaccinated goats showed typical disease symptoms in three among four controls vs. the challenged sheep (1/4). Goats have been preferred for challenges over sheep as the virulent strain was an isolate from this species, and several authors demonstrated a higher sensitivity of goats to mycoplasma infection [24]. It should be noted that sheep were challenged intratracheally according to Taoudi et al. [14], and goats were challenged via the SC route. 

To assess protection, vaccinated goats were challenged by an SC injection of the virulent Mcc MOR20 strain according to the challenge model proposed by Hasso et al. [15]. This challenge model demonstrated that this route of inoculation induces typical symptoms of disease including lameness, diarrhea, nasal secretions, anorexia, and keratoconjunctivitis. Two unvaccinated controls out of four presented high clinical scores with symptoms of hyperthermia, lameness, and keratoconjunctivitis. Two control animals did not present any symptoms or lesions due to mycoplasma, which is in accordance with observations reported by Sacchini et al. [25], who stated that only 50% of challenged animals may reproduce disease symptoms. All animals vaccinated with Mcc and Mccp vaccines tolerated the challenge with the Mcc virulent strain Mcc MOR20. These observations support the conclusion that the Mcc oil-adjuvant vaccine is efficient in protecting against Mcc infection. However, full cross-protection between Mcc and Mccp cannot be confirmed due to the lack of availability of a virulent strain of Mccp to challenge the Mcc-vaccinated animals. The serological response to vaccination was higher for Mccp than for Mcc, despite their equivalent levels of protection after a challenge with the pathogenic strain Mcc MOR20. According to Litamoi et al. [26], vaccination with the inactivated mycoplasma strain F38 reduced the number of goats presenting clinical symptoms of CA. The extent and effectiveness of cross-protection between mycoplasmas in that study can be explained by the genetic similarity of the subspecies, which shared the same virulence factors [2].

## 5. Conclusions

Mcc and Mccp share close genetic similarity, suggesting possible cross-serological responses and protection. We were not able to test cross-protection in this study, but both Mccp and Mcc vaccines provided protection when challenged with the virulent Mcc MOR20 strain. We were not able to test a challenge with a virulent Mccp strain. The inactivated vaccine with the oil adjuvant can provide robust protection against the infection and can be used at a large scale to protect small ruminants against CA and CCPP. 

## Figures and Tables

**Table 1:** Description of vaccine formulations.

Strain	Vaccine	Adjuvant	Type of Emulsion	Dose in 1.0 mL
Mcc MOR20	V1	Montanide	Water in oil	0.3 mg
	V2	Saponin	Aqueous solution	0.3 mg
	V3	Montanide	Oil in water	0.3 mg
Mccp F38	V4	Montanide	Water in oil	0.15 mg

**Table 2:** Groups of sheep vaccinated with *Mycoplasma capricolum* subsp. *capricolum* inactivated vaccines using three different adjuvants.

Vaccine	Species	Groups	Adjuvant	Animals	Route of Administration
Mcc Inactivated	Sheep	S1	Montanide W/O	4	IM
		S2	Saponin	4	SC
		S3	Montanide O/W	4	SC
Unvaccinated		S4	PBS	4	SC

**Table 3:** Groups of goats vaccinated with *Mycoplasma capricolum* subsp. *capricolum* and *Mycoplasma capricolum* subsp. *capripneumoniae* inactivated W/O vaccines.

Vaccine	Species	Groups	Adjuvant	Animals	Route of Administration
Mcc	Goat	G1	Montanide W/O	14	IM
Mccp		G2	Montanide W/O	14	IM
Unvaccinated		G3	PBS	4	SC

**Table 4:** Scoring of recorded clinical signs.

Clinical Signs		Score
General behavior	Normal	0
	Inactive	1
	Low head	2
	Very inactive	3
	Very inactive + hyperthermia	4
	Lying down	5
Respiratory symptoms	Normal	0
	Mucous nasal discharge	1
	Serous nasal discharge	2
	Purulent nasal discharge	3
	Nasal discharge + cough	4
	Nasal discharge + cough + dispnea	5
Digestive symptoms	Normal	0
	Soft diarrhea	1
	Liquid diarrhea	2
	Profuse diarrhea	3
	Liquid diarrhea + anorexia	4
	Profuse diarrhea + anorexia	5
Lameness and keratoconjonctivitis	Normal	0
	Lameness of 1 member	1
	Lameness of 2 members	2
	Keratoconjontivitis	3
	Polyarthritis	4
	Lameness + keratoconjonctivitis	5
Mortality		5

**Table 5:** Clinical scoring of vaccinated sheep challenged with Mcc.

Groupe	Sheep Number	General Behavior	Respiratory Symptoms	Digestive Symptoms	Lameness and Keratoconjonctivitis	Mortality	Score
Vaccinated Mcc W/O	746	1	0	0	0	0	1
	46	1	0	0	0	0	1
	731	1	0	0	0	0	1
	722	1	0	0	0	0	1
Vaccinated Mcc saponin	884	1	0	0	0	0	1
	871	0	0	0	1	0	1
	886	2	0	0	0	0	2
	842	0	2	0	0	0	2
Vaccinated Mcc O/W	745	5	2	2	3	0	12
	739	5	5	0	4	0	14
	788	0	0	0	1	0	1
	746	0	0	0	1	0	1
Unvaccinated	427	1	0	0	1	0	2
	748	3	2	0	0	0	5
	715	5	0	0	4	5	14
	714	3	0	0	2	0	5

**Table 6:** Clinical scoring of animals challenged with Mcc MOR20.

Group	Goat Number	General Behavior	Respiratory Symptoms	Digestive Symptoms	Lameness and Keratoconjonctivitis	Mortality	Score
Vaccinated Mcc G4	416	0	2	0	1	0	3
	422	2	3	0	0	0	5
	452	0	0	0	0	0	0
	426	2	2	0	0	0	4
Vaccinated Mccp G5	902	2	2	0	1	0	5
	909	1	0	0	1	0	2
	907	0	0	0	0	0	0
	914	0	2	0	0	0	2
Unvaccinated	933	5	4	5	4	5	23
	420	5	0	0	0	0	5
	405	5	4	5	5	5	24
	457	0	0	0	1	0	1

## Data Availability

All data analyzed during this study are included in this published article. All recorded raw data are archived at MCI Santé Animale.
